# PTS: a pharmaceutical target seeker

**DOI:** 10.1093/database/bax095

**Published:** 2017-12-28

**Authors:** Peng Ding, Xin Yan, Zhihong Liu, Jiewen Du, Yunfei Du, Yutong Lu, Di Wu, Yuehua Xu, Huihao Zhou, Qiong Gu, Jun Xu

**Affiliations:** Research Center for Drug Discovery, School of Pharmaceutical Sciences, Sun Yat-Sen University, Guangzhou 510006, China; National Supercomputer Center in Guangzhou, Sun Yat-Sen University, Guangzhou 510006, China and; School of Data and Computer Science, Sun Yat-Sen University, Guangzhou 510006, China

## Abstract

Identifying protein targets for a bioactive compound is critical in drug discovery. Molecular similarity is a main approach to fish drug targets, and is based upon an axiom that similar compounds may have the same targets. The molecular structural similarity of a compound and the ligand of a known target can be gauged in topological (2D), steric (3D) or static (pharmacophoric) metric. The topologic metric is fast, but unable to represent steric and static profile of a bioactive compound. Steric and static metrics reflect the shape properties of a compound if its structure were experimentally obtained, and could be unreliable if they were based upon the putative conformation data. In this paper, we report a pharmaceutical target seeker (PTS), which searches protein targets for a bioactive compound based upon the static and steric shape comparison by comparing a compound structure against the experimental ligand structure. Especially, the crystal structures of active compounds were taken into similarity calculation and the predicted targets can be filtered according to multi activity thresholds. PTS has a pharmaceutical target database that contains approximately 250 000 ligands annotated with about 2300 protein targets. A visualization tool is provided for a user to examine the result.

**Database URL**: http://www.rcdd.org.cn/PTS

## Introduction

For decades, the paradigm of drug discovery and development has been one-drug-for-one-target (1). Recent advances in systems biology (2) and chemical biology demonstrate that existing drugs can interact with multiple targets (3, 4). However, multi-target interactions are either unknown or insufficiently understood in most cases. There are increasing needs to predict drug targets for an agent due to growing number of bioactive compounds identified from phenotypic assays (5–7). The prediction has to be validated by experiments, such as structure biological approaches or proteomics. The *in silico* approaches can significantly reduce the costs and improve the performance of the experimental approaches for drug target fishing.

A drug target prediction method can be categorized into structure-based or ligand-based method. INDOCK (8) and TarFisDock (9) are typical structure-based target fishing tools using molecular docking algorithms, which rely on the target structure availability and the structure diversity of the binding pocket. However, a ligand-based target fishing approach uses the ligand-compound similarity based on topological structures (fingerprints) (10, 11), molecular shapes, pharmacophores (12) or compound activity profiles (13). The ligand-based target fishing approaches are being adopted due to the increasing availability of bioassay data (14–16). SEA (17) and SuperPred (18) are typical ligand-based approaches that use ligand databases and compound topological (2D) similarity measurements. Other methods, such as Chemmapper (19), Superimpose (20) and wwLigCSRre (21) use 3D structure similarity metric to predict protein targets. 2D and 3D similarity measurements are complimentary, and 3D similarity measurements seem capable of picking novel chemotypes (22) if the template structures were experimentally obtained.

In this work, we have implemented a pharmaceutical target seeker (PTS), which uses the experimental 3D structures of ligands with known targets to calculate the similarity of the ligand and a compound. For those ligands for which experimental structure data are not available, their energy-minimized conformations are generated for the 3D similarity calculations. The 3D similarity search engine is Weighted Gaussian Algorithm (WEGA) (23), which can take steric and pharmacophoric profile into account. The user can rule out impossible targets by setting activity thresholds in order to expedite the target fishing process. PTS contains approximately 250 000 ligands annotated with 2300 protein targets.

## Materials and methods

### Data preparation

The data of bioactive compounds and their targets were collected from public databases.

Target data were derived from therapeutic target database (TTD version 2015) (24) and reference (25). Through UniProt ID, ligand data and their relations with targets were extracted from UniProt (26), ChEMBL20 (27) and BindingDB (28, 29), PDBbind (version 2014) (30–32) and RCSB PDB databases.

The data were pre-processed with the following steps:

1. removing obsolete UniProt IDs from TTD target data;
2. removing counter ion moieties from bioactive ligands;
3. removing compounds from ChEMBL20 data if their activity (IC50/Ki/Kd) values are greater than 50 μM;
4. removing small compounds (heavy atoms < 6) and large compounds (MW > 1000 Da).

This resulted in 266 866 ligands associated with 2298 protein targets, 537 095 bioactivity data points, 4391 crystal structures and 16 590 related articles in the PTS built-in database (Table 1). Among the targets, 14% of them have drugs in the market, 41% of them have drug candidates under clinic trails, 40% of them have ligands under the investigations and 5% of them have compounds that were discontinued for pharmaceutical studies.

**Table 1.** Statistics data of PTS

Data	Number	Source	Number	Availability
	(extracted)		(original)	
Target	2298	TTD (2015)	2875	bidd.nus.edu.sg/group/cjttd/
Compound	266 866	ChEMBL20	1 463 270	www.ebi.ac.uk/chembl/
PDB	4391	PDBbind (2014)	10 656	www.pdbbind-cn.org/
Activity record	537 095	ChEMBL20	13 520 737	www.ebi.ac.uk/chembl/
Reference	16 590	ChEMBL20	59 610	www.ebi.ac.uk/chembl/

### Similarity algorithm

The target fishing process is based upon an axiom that similar compounds may have the same targets. An in-house algorithm, WEGA, is used to compute the steric and static similarity of a ligand-compound pair. WEGA is based on the first order Gaussian approximation, which simplifies the shape density functions of the molecules by avoiding expensive higher order terms calculation.

WEGA offers three similarity calculation methods: 1) shape matching, which is only the molecular volumetric overlay, 2) feature matching, which is the pharmacophore mapping of molecule pair, 3) combination matching, which integrates the advantage of the above two aspects and is the most precise approach. The detailed method of WEAG is described in reference (23).

### Webserver implementation

PTS uses a browser and server framework. Client interface was implemented in HTML and Javascript. The back-end is implemented in Golang language and MySQL database system. The molecule editor and chemical structure viewers are supported with Marvin JS and ChemDoodle web component. All tools have been summarized in Table 2.

**Table 2.** The tools used for PTS implementation

Tool	Use	Availability
Marvin JS	Chemical structure input	marvinjs-demo.chemaxon.com/latest/
ChemDoodle Web	Structure draw and 3D display	web.chemdoodle.com/
Open Babel	Chemical file format conversion	openbabel.org/wiki/Main_Page
Discovery Studio	3D conformation generation	accelrys.com/products/collaborative-science/biovia-discovery-studio/
MySQL	Storage database	www.mysql.com/
JQuery	Foreground and background interaction	jquery.com/
Golang	Web server language	golang.org/

## Results

### Workflow

**Figure 1.**
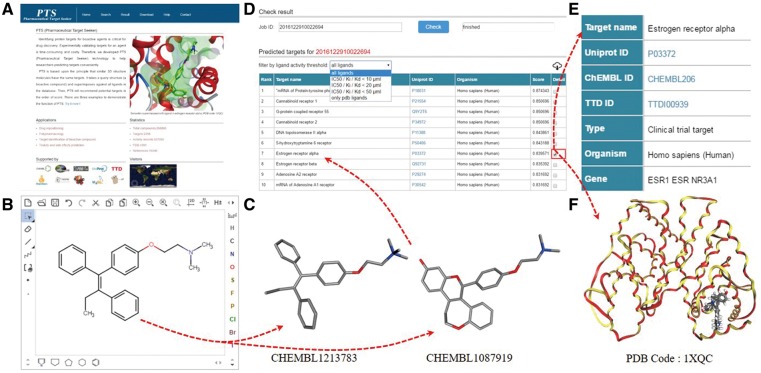
PTS working protocol. (**A**) Main menu, (**B**) chemical structure editor, (**C**) ligand structure from PTS builtin ligand database, (**D**) predicted target list, (**E**) target profile and (**F**) query molecules docked in the binding pocket of a predicted target.

PTS provides an intuitive interface to predict small molecule protein targets. A user can input a query molecule by uploading a file (mol/SDF format) or drawing a chemical structure with its built-in chemical structure editor (Figure 1B). PTS will generate the possible 3D conformations for the query and, employ WEGA to compute the 3D similarities of the molecular conformations and the ligand structures in PTS ligand database (Figure 1C). A typical task of PTS takes about 30–60 min, depending on the flexibility of the query compound and calculation method assigned by users. Each user’s query is automatically assigned with a Job-ID that allows the user to receive and inspect the target prediction result (Figure 1D). The result page lists the predicted targets with their common names linked to UniProt (26) database if they are available. Targets are ranked according to their score with respect to the query molecule. Thus, the most possible target is placed at the top of the list. Sometimes, multiple targets may be inferred for the query compound based on a single similar compound. If so, the order of the target presented in the table has no specific meaning regarding to prediction significance. Besides, predicted targets can be tailored by the activity threshold of a template ligand (such as 10, 20 or 50 µM) (Figure 1D).

By clicking on the check button, a user can inspect the predicted template ligands for a query molecule (Figure 1D). In the result page, target data fields include UniProt ID, CHEMBL ID, TTD ID, type, organism, gene and biological functions (Figure 1E). The template ligands are ranked based upon their similarity values to the query molecule. The resulting targets can be tailored by setting the 3D similarity values to the query molecule (default threshold is 0.6, and ligands show low similarity with the query if below this threshold). For the template ligands with experimental structure data, the query molecular structure is superimposed with the ligands in the binding pocket of the predicted target (Figure 1F), and downloadable.

### Case study 1: seeking targets for Afatinib

Afatinib is an irreversible kinase inhibitor targeting epidermal growth factor receptor (EGFR) and inhibiting tyrosine kinase auto-phosphorylation (33) to stop tumor cells growth. Afatinib is available for the first-line treatment of patient with metastatic non-small cell lung cancer. The targets predicted by PTS are listed in Table 3.

**Table 3.** The predicted targets for Afatinib

Rank	UniProt ID	Target name	Organism	Score
1	P00533	EGFR	*Homo sapiens* (Human)	0.74
2	P25440	Bromodomain-containing protein 2	*Homo sapiens* (Human)	0.72
3	Q15059	Bromodomain-containing protein 3	*Homo sapiens* (Human)	0.72
4	O60885	Bromodomain-containing protein 4	*Homo sapiens* (Human)	0.72
5	P34969	5-hydroxytryptamine 7 receptor	*Homo sapiens* (Human)	0.72
6	Q07820	Induced myeloid leukemia cell differentiation protein Mcl-1	*Homo sapiens* (Human)	0.72
7	P09917	mRNA of human 5-lipoxygenase	*Homo sapiens* (Human)	0.72
8	P17948	Vascular endothelial growth factor receptor 1	*Homo sapiens* (Human)	0.72
9	P08253	72 kDa type IV collagenase	*Homo sapiens* (Human)	0.71
10	P24557	Thromboxane-A synthase	nil	0.71

**Figure 2.**
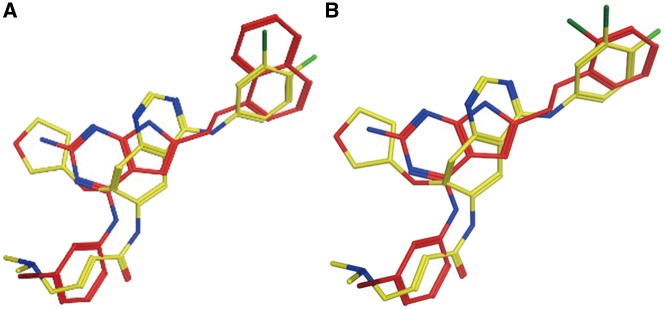
Afatinib (yellow) aligned with known EGFR inhibitor CHEMBL484108 (**A**, red) and CHEMBL482489 (**B**, red).

Experimental data indicate that Afatinib is an EGFR inhibitor (IC50 = 1 nM) (34). EGFR (UniProt ID: P00533) is ranked at the top of the predicted target list by PTS (Table 3). The predicted Afatinib binding poses are aligned with the native EGFR ligands as shown in Figure 2. PTS also predicted other potential targets, however, there are no evidences showing that Afatinib is strongly binding with them. The data for the alignments of Afatinib and the native ligands of these targets can be found in Supplementary Figure S1.

### Case study 2: seeking targets for Tamoxifen

**Figure 3.**
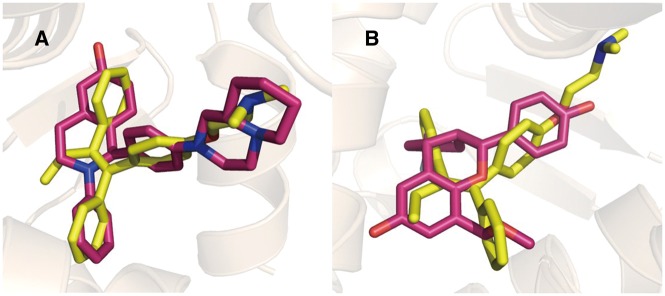
Tamoxifen (yellow) aligned with ERα (**A**, red, PDB Code: 1XQC) and ERβ (**B**, red, PDB Code: 2QTU) selective ligands in binding pocket.

Estrogen receptors (ERs) are well-known targets for Tamoxifen (35–37). The targets predicted by PTS for tamoxifen are listed in Table 2. ERα (UniProt ID: P03372) and ERβ (UniProt ID: Q92731) are ranked as the top-1 and top-6. The predicted Tamoxifen binding poses aligned with the native ERα and ERβ ligands are depicted in Figure 3.

In Table 4, PTS places top-10 targets. After examing the other predicted targets besides ERs, we find that CYP2D6 (38), Prostaglandin synthase (40), 3-β-hydroxysteroid-δ(8),δ(7)-isomerase (39) and Collagenase 3 (41, 42) has been experimentally reported as off-targets. In fact, 38 proteins have been experimentally validated to interact with tamoxifen or 4 H-tamoxifen (Supplementary Table S1). In the top-100 PTS predicted targets for Tamoxifen, 15% proteins are experimentally confirmed off-targets, which can be found in Supplementary Table S2.

**Table 4.** Experimentally proved Tamoxifen targets and off-targets predicted by PTS

Rank	UniProt ID	Target name	Organism	Score
1	P03372	ER alpha	*Homo sapiens* (Human)	0.82
2	P04035	3-hydroxy-3-methylglutaryl-coenzyme A reductase	*Homo sapiens* (Human)	0.82
3	P08684	mRNA of CYP3A4	*Homo sapiens* (Human)	0.82
4	P23458	JAK1	*Homo sapiens* (Human)	0.81
5	P41145	Kappa-type opioid receptor	*Homo sapiens* (Human)	0.81
6	Q92731	ER beta	*Homo sapiens* (Human)	0.81
7	O14965	Aurora kinase A	*Homo sapiens* (Human)	0.80
8	Q96GD4	Serine/threonine protein kinase 12	*Homo sapiens* (Human)	0.80
9	P29597	TYK2	*Homo sapiens* (Human)	0.80
10	P52333	Tyrosine-protein kinase JAK3	*Homo sapiens* (Human)	0.80
11	P10635	Cytochrome P450 2D6	*Homo sapiens* (Human)	0.80

### Case study 3: validating a target for Chlorprothixene

**Figure 4.**
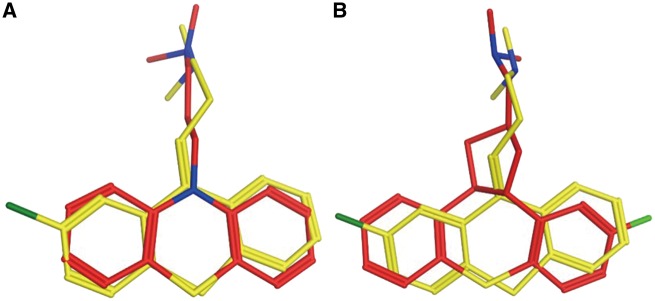
Chlorprothixene (red) aligned with H1 receptor antagonist promazine (**A**, yellow) and CHEMBL363581 (**B**, yellow).

Chlorprothixene is an old antipsychotic drug. It antagonizes dopaminergic D1 (UniProt ID: P21728) and D2 (UniProt ID: P14416) receptors in the brain to exert its antipsychotic effect (43). Chlorprothixene also antagonizes histamine H1 receptor (44). But, there is no direct evidence to show Chlorprothixene interacts with H1. PTS predicts H1 receptor is the target of Chlorprothixene, the results are listed in Supplementary Table S3 (Figure 4). Sridhar R. Vasudevan proved experimentally that Chlorprothixene binds with H1 receptor and selectively inhibit histamine-induced calcium release with IC50 of 1 nM.

### Case study 4: predicting potential side-effects for Fluoxetine

hERG (the human Ether-à-go-go-Related Gene, UniProt ID: Q12809) is known as a potassium (K+) ion channel mediating the repolarizing IKr current in the cardiac action potential. A drug that potentially interacts with hERG can result in lethal side-effect (45). Fluoxetine is a selective serotonin reuptake inhibitor for treating depressive disorder. The potential targets PTS predicted for Fluoxetine are listed in Table 5.

**Table 5.** The predicted target profile for fluoxetine

Rank	UniProt ID	Target name	Organism	Score
1	P35354	Prostaglandin G/H synthase 2	*Homo sapiens* (Human)	0.86
2	P23219	Prostaglandin G/H synthase 1	*Homo sapiens* (Human)	0.86
3	Q01959	Sodium-dependent dopamine transporter	*Homo sapiens* (Human)	0.85
4	P31645	Sodium-dependent serotonin transporter	*Homo sapiens* (Human)	0.85
5	P23975	Sodium-dependent noradrenaline transporter	*Homo sapiens* (Human)	0.85
6	P14416	D(2) dopamine receptor	*Homo sapiens* (Human)	0.84
7	P35462	D(3) dopamine receptor	*Homo sapiens* (Human)	0.84
8	P11229	Muscarinic receptor	*Homo sapiens* (Human)	0.83
9	P35367	Histamine H1 receptor	*Homo sapiens* (Human)	0.83
10	Q12809	Potassium channel H-ERG	*Homo sapiens* (Human)	0.83

Sodium-dependent serotonin transporter (UniProt ID: P31645) is the primary target of Fluoxetine, which is ranked at fourth in the list. By inspect the potential target list, we find the top-10 target is hERG. Further literature studies reveal that Fluoxetine is experimentally proved as hERG inhibitor (IC50 = 3.1 μM) (46).

## Discussion

PTS predicts targets for a compound through superimposing the compound structure onto the 3D ligand structures of putative targets. This approach considers pharmacophore shape similarity that 2D approaches cannot do. Other 3D approaches use molecular docking techniques, while PTS does not employ ligand-receptor docking techniques and can still produce results where the receptor structure data are not available. Both PTS and SwissTargetPrediction are web-based target fishing tools. The four testing cases were tested on both tools, which produced similar results. Additionally, small scale of structurally diverse drugs that targeting four classes of biological systems (GPCR, Ion channel, Nuclear receptor and kinase) were extracted to test the success rate and applicability of PTS. Averagely, at least one known targets of each drug is found among the top 20 predicted targets for 70% of the ligands (Supplementary Material). The advantage of PTS is able to superimpose the query molecule into the binding pockets of the putative targets when the experimental structure data are available. However, there are limits for PTS, too. The activity cliff issue, i.e. a subtle change in the chemical structure cause the great loss of bioactivity, may be a common concern for all ligand-based methods, including the 3D approaches.

To date, PTS has received >500 queries from 11 countries in the world since 10 August 2016. It can be used for target identification, drug repurposing, toxic risk estimation and molecular interaction simulation pre-processing tool.

## Funding

National Science Foundation of China (81473138, 81573310); Guangdong Frontier & Key Technology Innovation Program (2015B010109004); Guangdong Natural Science Foundation (2016A030310228); the Fundamental Research Funds for the Central Universities (2013HGCH0015); Tianhe-2 based biomedical health data application support platform (U1611261); the Fundamental Research Funds for the Central Universities (17LGJC23). Funding for open access charge: National Science Foundation of China (81473138), funded by the National Natural Science Foundation of China.

## Supplementary data

Supplementary data are available at *Database* Online.

*Conflict of interest.* None declared.

## Supplementary Material

Supplementary Data
