# Supplementary material for: PTS: a pharmaceutical target seeker
Source: Database (Oxford). 2017 Dec 28;2017:bax095. doi: 10.1093/database/bax095 (PMC5750839; doi:10.1093/database/bax095)
Supplement: Supplementary Data [file bax095_supp.docx]

**Supplementary materials**

**Case Study 1: Seeking targets for Afatinib**

PTS predicted other potential targets for Afatinib. However, there are no evidences showing that Afatinib is strongly binding with them. Here is an example of the alignments of Afatinib and the native ligands of Tyrosine-protein kinase LCK.

Job parameter settings:

Chemical type: 2D structure

Screening method: shape

Similary threshold: 0.6


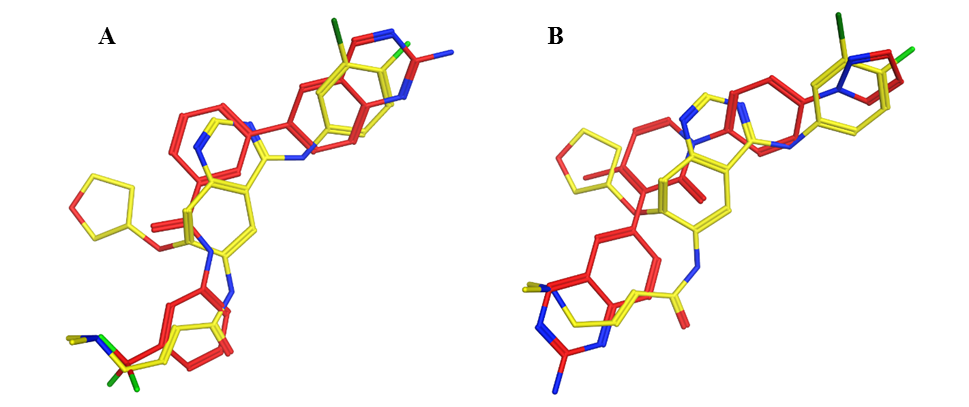
**Fig 1.** Afatinib (yellow) aligned with known Tyrosine-protein kinase LCK inhibitor CHEMBL215529 (A, red) and CHEMBL497343 (B, red).

**Case Study 2: Seeking targets for Tamoxifen**

Job parameter settings:

Chemical type: 2D structure

Screening method: shape

Similary threshold: 0.6

**Table S1 The experimentally validated binding protein targets of tamoxifen**

| Target Name | Reference |
| --- | --- |
| Estrogen receptor | ([1](#_ENREF_1)) |
| Estrogen receptor beta | ([2](#_ENREF_2)) |
| Sulfotransferase 1A1 | ([3](#_ENREF_3)) |
| ATP-binding cassette sub-family G member 2 | ([4](#_ENREF_4)) |
| Dimethylaniline monooxygenase [N-oxide-forming] 3 | ([5](#_ENREF_5)) |
| Canalicular multispecific organic anion transporter 1 | ([6](#_ENREF_6)) |
| Dimethylaniline monooxygenase [N-oxide-forming] 1 | ([5](#_ENREF_5)) |
| Multidrug resistance protein 1 | ([7](#_ENREF_7)) |
| Bile salt export pump | ([8](#_ENREF_8)) |
| 3-beta-hydroxysteroid-Delta(8),Delta(7)-isomerase | ([9](#_ENREF_9)) |
| Protein kinase C | ([10](#_ENREF_10)) |
| Liver carboxylesterase 1 | ([11](#_ENREF_11)) |
| UDP-glucuronosyltransferase 1-4 | ([12](#_ENREF_12)) |
| Cytochrome P450 19A1 | ([13](#_ENREF_13)) |
| Cytochrome P450 1A1 | ([5](#_ENREF_5)) |
| Cytochrome P450 1A2 | ([5](#_ENREF_5)) |
| Cytochrome P450 1B1 | ([14](#_ENREF_14)) |
| Cytochrome P450 2A6 | ([13](#_ENREF_13)) |
| Cytochrome P450 2B6 | ([15](#_ENREF_15)) |
| Cytochrome P450 2C8 | ([16](#_ENREF_16)) |
| Cytochrome P450 2C9 | ([5](#_ENREF_5)) |
| Cytochrome P450 2C19 | ([15](#_ENREF_15)) |
| Cytochrome P450 2D6 | ([17](#_ENREF_17)) |
| Cytochrome P450 2E1 | ([13](#_ENREF_13)) |
| Cytochrome P450 3A4 | ([18](#_ENREF_18)) |
| Cytochrome P450 3A5 | ([5](#_ENREF_5)) |
| Cytochrome P450 3A7 |  |
| 17β-hydroxysteroid dehydrogenase | ([19](#_ENREF_19)) |
| Dihydrofolate reductase | ([20](#_ENREF_20)) |
| Glutathione transferase | ([21](#_ENREF_21)) |
| Prostaglandin synthase | ([22](#_ENREF_22)) |
| Collagenase | ([23](#_ENREF_23)) |
| 3α-hydroxysteroid dehydrogenase | ([24](#_ENREF_24)) |
| Calmodulin | ([2](#_ENREF_2)) |
| Alcohol dehydrogenase | ([25](#_ENREF_25)) |
| Immunoglobulin | ([26](#_ENREF_26)) |
| Microsomal antiestrogen binding site (AEBS) | ([2](#_ENREF_2)) |
| Cholesterol Acyl Transferase (ACAT) | ([2](#_ENREF_2)) |

**Table S2 100 potential targets of tamoxifen predicted by PTS**

| Rank | Score | UniProt_ID | Target_name |
| --- | --- | --- | --- |
| 1 | 0.824571 | P03372 | Estrogen receptor alpha |
| 2 | 0.819303 | P04035 | 3-hydroxy-3-methylglutaryl-coenzyme A reductase |
| 3 | 0.81577 | P08684 | mRNA of CYP3A4 |
| 4 | 0.813385 | P23458 | JAK1 |
| 5 | 0.808493 | P41145 | Kappa-type opioid receptor |
| 6 | 0.808096 | Q92731 | Estrogen receptor beta |
| 7 | 0.804358 | O14965 | Aurora kinase A |
| 8 | 0.804358 | Q96GD4 | Serine/threonine protein kinase 12 |
| 9 | 0.803884 | P29597 | TYK2 |
| 10 | 0.803884 | P52333 | Tyrosine-protein kinase JAK3 |
| 11 | 0.800854 | P10635 | Cytochrome P450 2D6 |
| 12 | 0.79411 | P24557 | Thromboxane-A synthase |
| 13 | 0.788913 | Q15858 | Voltage-gated sodium channel subunit alpha Nav1.7 |
| 14 | 0.78804 | Q16539 | Mitogen-activated protein kinase 14 |
| 15 | 0.78133 | P08172 | Muscarinic receptor |
| 16 | 0.78133 | P31645 | Sodium-dependent serotonin transporter |
| 17 | 0.78133 | P13945 | Beta-3 adrenergic receptor |
| 18 | 0.78133 | P18089 | Alpha-2B adrenergic receptor |
| 19 | 0.78133 | P23975 | Sodium-dependent noradrenaline transporter |
| 20 | 0.78133 | P11229 | Muscarinic receptor |
| 21 | 0.78133 | P50406 | 5-hydroxytryptamine 6 receptor |
| 22 | 0.78133 | P20309 | Muscarinic receptor |
| 23 | 0.78133 | P08173 | Muscarinic receptor |
| 24 | 0.78133 | P08912 | Muscarinic receptor |
| 25 | 0.78133 | P08913 | Alpha 2 adrenoceptor |
| 26 | 0.78133 | P18825 | Alpha 2 adrenoceptor |
| 27 | 0.78133 | Q15125 | Cholestenol delta-isomerase |
| 28 | 0.78133 | P00533 | mRNA of EGFR |
| 29 | 0.78133 | P06241 | Fyn tyrosine kinase |
| 30 | 0.78133 | P04626 | Erbb2 tyrosine kinase receptor |
| 31 | 0.78133 | P35462 | D(3) dopamine receptor |
| 32 | 0.78133 | Q01959 | Sodium-dependent dopamine transporter |
| 33 | 0.78133 | P23219 | Prostaglandin G/H synthase 1 |
| 34 | 0.78133 | P21452 | Substance-K receptor |
| 35 | 0.78133 | P28223 | 5-hydroxytryptamine 2A receptor |
| 36 | 0.78133 | P41595 | 5-hydroxytryptamine 2B receptor |
| 37 | 0.78133 | P28335 | 5-hydroxytryptamine 2C receptor |
| 38 | 0.78133 | Q99720 | Sigma(1)-type opioid receptor |
| 39 | 0.780486 | P35372 | Peripherally-acting muopioid receptor |
| 40 | 0.780486 | P41146 | Nociceptin receptor |
| 41 | 0.780486 | P41143 | Delta-type opioid receptor |
| 42 | 0.778731 | O14757 | Serine/threonine-protein kinase Chk1 |
| 43 | 0.777639 | Q9Y233 | Phosphodiesterase 10A (PDE10A) |
| 44 | 0.774929 | P11511 | Cytochrome P450 19 |
| 45 | 0.773947 | Q07912 | Activated CDC42 kinase 1 |
| 46 | 0.771006 | P18031 | mRNA of Protein-tyrosine phosphatase, non-receptor type 1 |
| 47 | 0.770212 | Q13526 | Peptidyl-prolyl cis-trans isomerase NIMA-interacting 1 |
| 48 | 0.769506 | P04150 | mRNA of glucocorticoid receptor |
| 49 | 0.767304 | P37288 | Vasopressin V1a receptor |
| 50 | 0.767304 | P30559 | Oxytocin receptor |
| 51 | 0.76724 | P22001 | Potassium voltage-gated channel subfamily A member 3 |
| 52 | 0.765533 | Q9Y5Y4 | Putative G-protein coupled receptor 44 |
| 53 | 0.765351 | P43235 | Cathepsin K |
| 54 | 0.764442 | P35354 | Prostaglandin G/H synthase 2 |
| 55 | 0.763358 | P31749 | RAC-alpha serine/threonine kinase; AkT |
| 56 | 0.763214 | P06756 | Integrin alpha-V/beta-5 |
| 57 | 0.761621 | Q8TDV5 | Glucose-dependent insulinotropic receptor |
| 58 | 0.761284 | P14416 | D(2) dopamine receptor |
| 59 | 0.761284 | P21917 | D(4) dopamine receptor |
| 60 | 0.761213 | P21554 | Cannabinoid receptor 1 |
| 61 | 0.761213 | Q9Y2T6 | G-protein coupled receptor 55 |
| 62 | 0.760357 | P29466 | Interleukin-1 beta convertase |
| 63 | 0.757892 | P12931 | Src tyrosine kinase |
| 64 | 0.757892 | P06239 | Lck tyrosine kinase |
| 65 | 0.757892 | P00519 | mRNA of Abl |
| 66 | 0.757892 | P29320 | Epha3 tyrosine kinase receptor |
| 67 | 0.75781 | P00918 | Carbonic anhydrase II |
| 68 | 0.757516 | P00734 | Thrombin |
| 69 | 0.756814 | P49841 | Glycogen synthase kinase-3 beta |
| 70 | 0.756814 | P49840 | Glycogen synthase kinase-3 |
| 71 | 0.75676 | Q01726 | Melanocyte stimulating hormone receptor |
| 72 | 0.75676 | P41968 | Melanocortin-3 receptor |
| 73 | 0.75676 | P32245 | Melanocortin-4 receptor |
| 74 | 0.756583 | P06401 | Progesterone receptor |
| 75 | 0.756583 | P10275 | mRNA of androgen receptor |
| 76 | 0.756525 | P21462 | N-formyl peptide receptor |
| 77 | 0.756382 | Q13946 | Phosphodiesterase (PDE) 7 |
| 78 | 0.756185 | P46663 | Bradykinin receptor |
| 79 | 0.755236 | Q9HC97 | G protein-coupled receptor 35 |
| 80 | 0.754901 | P09917 | mRNA of human 5-lipoxygenase |
| 81 | 0.753666 | P48067 | Sodium- and chloride-dependent glycine transporter 1 |
| 82 | 0.752584 | P15144 | Aminopeptidase N |
| 83 | 0.751201 | Q9NR96 | Toll-like receptor 9 |
| 84 | 0.750541 | P45452 | Collagenase 3 |
| 85 | 0.750522 | P25929 | Neuropeptide Y receptor type 1 |
| 86 | 0.750522 | P49146 | Neuropeptide Y receptor type 2 |
| 87 | 0.749794 | P47901 | Vasopressin V1b receptor |
| 88 | 0.749794 | P30518 | Vasopressin V2 receptor |
| 89 | 0.749221 | P39900 | Macrophage metalloelastase |
| 90 | 0.749221 | P08253 | 72 kDa type IV collagenase |
| 91 | 0.749221 | P14780 | 92 kDa type IV collagenase |
| 92 | 0.749164 | Q8TDU6 | TGR5 |
| 93 | 0.749116 | P07339 | Cathepsin D |
| 94 | 0.747689 | P13922 | Dihydrofolate reductase |
| 95 | 0.747558 | Q9Y5N1 | Histamine H3 receptor |
| 96 | 0.74665 | O60674 | Tyrosine-protein kinase JAK2 |
| 97 | 0.746322 | Q96RI1 | Bile acid receptor |
| 98 | 0.746061 | P08908 | Serotonin-1A |
| 99 | 0.746021 | P36544 | Nicotinic ACh receptor |
| 100 | 0.74592 | O43570 | Carbonic anhydrase XII |

**Case study 3: Validating a target for Chlorprothixene**

Job parameter settings:

Chemical type: 2D structure

Screening method: shape

Similary threshold: 0.6

**Table S3 50 potential protein targets of chlorprothixene predicted by PTS**

| Rank | Score | UniProt_ID | Target_name |
| --- | --- | --- | --- |
| 1 | 0.900264 | Q99571 | P2X purinoceptor 4 |
| 2 | 0.891521 | P06241 | Fyn tyrosine kinase |
| 3 | 0.891521 | P50406 | 5-hydroxytryptamine 6 receptor |
| 4 | 0.891521 | P31645 | Sodium-dependent serotonin transporter |
| 5 | 0.891521 | P21917 | D(4) dopamine receptor |
| 6 | 0.891521 | P18089 | Alpha-2B adrenergic receptor |
| 7 | 0.891521 | P23975 | Sodium-dependent noradrenaline transporter |
| 8 | 0.891521 | P11229 | Muscarinic receptor |
| 9 | 0.891521 | P08172 | Muscarinic receptor |
| 10 | 0.891521 | P20309 | Muscarinic receptor |
| 11 | 0.891521 | P08173 | Muscarinic receptor |
| 12 | 0.891521 | P08912 | Muscarinic receptor |
| 13 | 0.891521 | P08913 | Alpha 2 adrenoceptor |
| 14 | 0.891521 | P18825 | Alpha 2 adrenoceptor |
| 15 | 0.891521 | P25100 | Alpha 1 adrenoceptor |
| 16 | 0.891521 | P33032 | Melanocortin MC5 receptor |
| 17 | 0.891521 | P34969 | 5-hydroxytryptamine 7 receptor |
| 18 | 0.891521 | P04156 | Major prion protein |
| 19 | 0.891521 | Q12809 | Potassium channel H-ERG |
| 20 | 0.891521 | P08908 | Serotonin-1A |
| 21 | 0.891521 | P35372 | Peripherally-acting muopioid receptor |
| 22 | 0.891521 | P10635 | Cytochrome P450 2D6 |
| 23 | 0.891521 | P21452 | Substance-K receptor |
| 24 | 0.891521 | P21728 | Dopamine D1 receptor |
| 25 | 0.891521 | P14416 | D(2) dopamine receptor |
| 26 | 0.891521 | P35462 | D(3) dopamine receptor |
| 27 | 0.891521 | P21918 | D(1B) dopamine receptor |
| 28 | 0.891521 | Q01959 | Sodium-dependent dopamine transporter |
| 29 | 0.891521 | P08183 | Multidrug resistance protein 1 |
| 30 | 0.891521 | P35367 | Histamine H1 receptor |
| 31 | 0.891521 | P25021 | Histamine H2 receptor |
| 32 | 0.891521 | P28223 | 5-hydroxytryptamine 2A receptor |
| 33 | 0.891521 | P41595 | 5-hydroxytryptamine 2B receptor |
| 34 | 0.891521 | P28335 | 5-hydroxytryptamine 2C receptor |
| 35 | 0.891521 | P41145 | Kappa-type opioid receptor |
| 36 | 0.891521 | P41143 | Delta-type opioid receptor |
| 37 | 0.891521 | Q99720 | Sigma(1)-type opioid receptor |
| 38 | 0.891521 | P62158 | Calmodulin |
| 39 | 0.891521 | Q06278 | Aldehyde oxidase |
| 40 | 0.881329 | P11309 | Proto-oncogene pim-1 |
| 41 | 0.881329 | Q9P1W9 | Serine/threonine-protein kinase pim-2 |
| 42 | 0.87851 | Q07820 | Induced myeloid leukemia cell differentiation protein Mcl-1 |
| 43 | 0.876182 | P09917 | mRNA of human 5-lipoxygenase |
| 44 | 0.876069 | P03372 | Estrogen receptor alpha |
| 45 | 0.876069 | Q92731 | Estrogen receptor beta |
| 46 | 0.875542 | Q9Y2T6 | G-protein coupled receptor 55 |
| 47 | 0.871297 | Q9Y233 | Phosphodiesterase 10A (PDE10A) |
| 48 | 0.868335 | P00390 | Glutathione reductase |
| 49 | 0.86488 | P22303 | Acetylcholinesterase |
| 50 | 0.863577 | P16083 | NRH dehydrogenase [quinone] 2 |

**Case Study 4: Predicting potential side-effects for Fluoxetine**

Job parameter settings:

Chemical type: 2D structure

Screening method: shape

Similary threshold: 0.6

**Comparison result with other software**

1. Predicted targets of Afatinib by SwissTargetPrediction


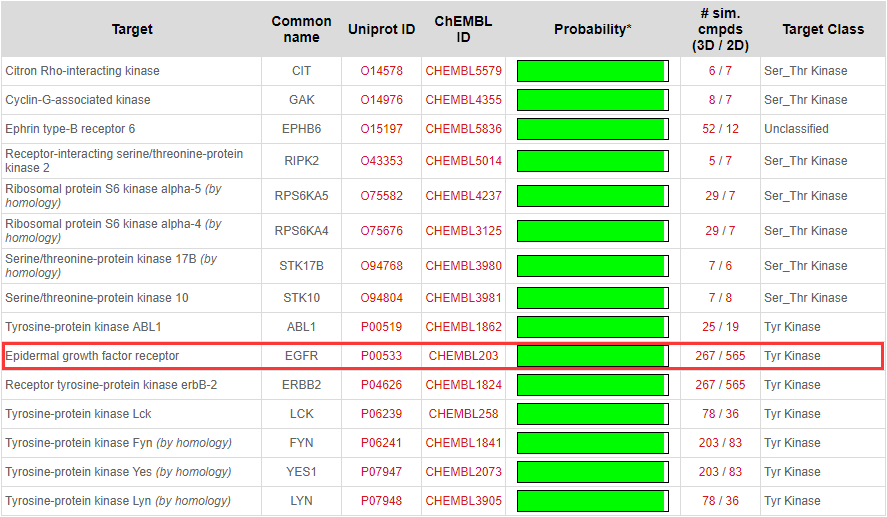


2. Predicted targets of Tamoxifen by SwissTargetPrediction


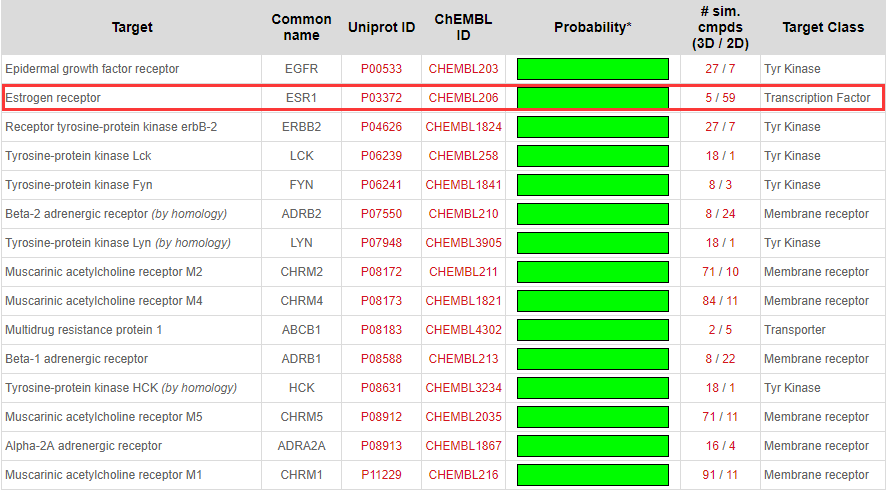


3. Predicted targets of Chlorprothixene by SwissTargetPrediction


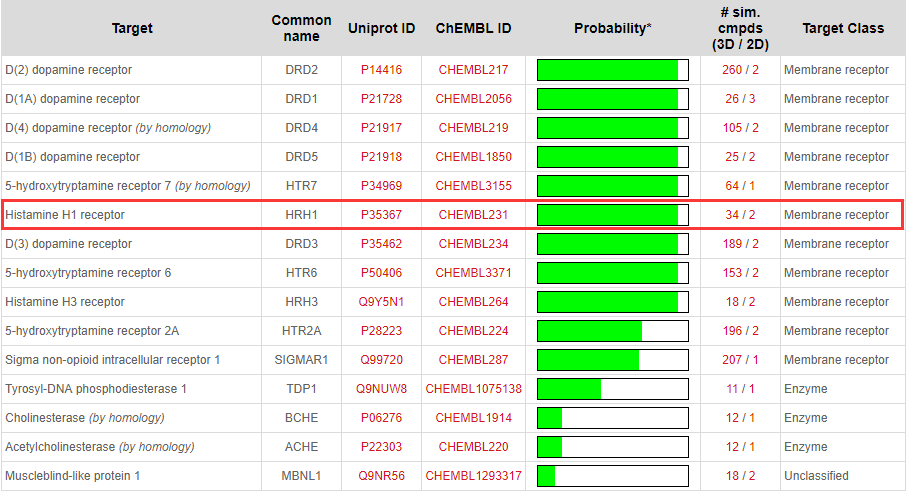


4. Predicted targets of Fluoxetine by SwissTargetPrediction


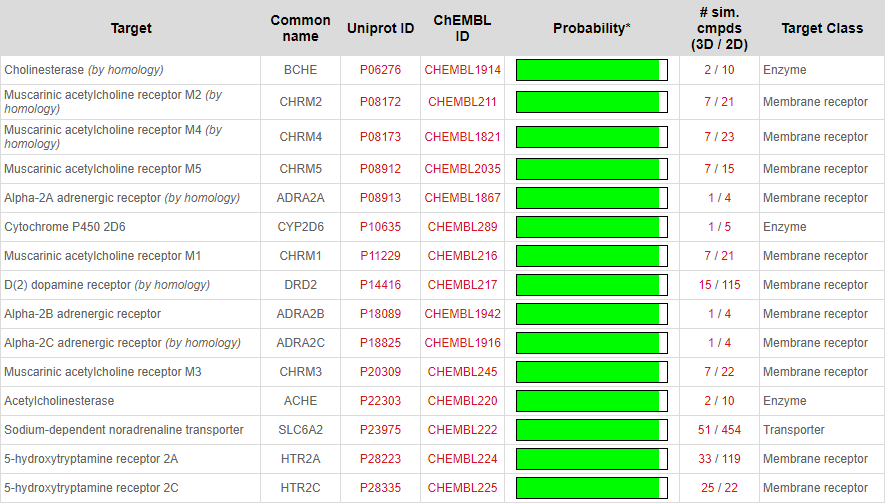


**The success rate and applicability test of PTS**

Small scale of structurally diverse drugs that distributing in four classes (GPCR, Ion channel, Nuclear receptor and kinase) were extracted to test the success rate and applicability of our software. The job parameter settings are same as above four test cases and test results are shown as follows.

**Table S4.** The success rate and applicability test of PTS.

| Target classes | Number of test drugs | Ratio (top-20) | Ratio (top-100) |
| --- | --- | --- | --- |
| GPCR | 10 | 70% | 90% |
| Ion channel | 10 | 60% | 70% |
| Nuclear receptor | 10 | 80% | 80% |
| Enzyme | 10 | 70% | 90% |
| Total | 40 | 70% | 82.5% |

**Table S5.** The detail targets and drugs information of the success rate and applicability test of PTS.

| **Target class** | **Target name** | **UniProt ID** | **Drug name** | **PTS ranking** |
| --- | --- | --- | --- | --- |
| GPCR | Adenosine receptor A1 | P30542 | Caffeine CHEMBL113 | 10 |
| GPCR | 5-hydroxytryptamine receptor 2A | P28223 | Cabergoline CHEMBL1201087 | 18 |
| GPCR | 5-hydroxytryptamine receptor 2B | P41595 | Eletriptan CHEMBL1510 | 31 |
| GPCR | Beta-2 adrenergic receptor | P07550 | Arformoterol CHEMBL1363 | 306 |
| GPCR | D(2) dopamine receptor | P14416 | Amisulpride CHEMBL243712 | 46 |
| GPCR | Dopamine D1 receptor | P21728 | Chlorprothixene CHEMBL908 | 5 |
| GPCR | Gamma-aminobutyric acid B receptor | Q9UBS5 | Propofol CHEMBL526 | 3 |
| GPCR | Histamine H3 receptor | Q9Y5N1 | Histamine CHEMBL90 | 1 |
| GPCR | Muscarinic receptor | P20309 | Disopyramide CHEMBL517 | 4 |
| GPCR | Sodium-dependent serotonin transporter | P31645 | Imipramine CHEMBL11 | 3 |
| Ion channel | 5-hydroxytryptamine receptor 3A | P46098 | Amoxapine CHEMBL1113 | 14 |
| Ion channel | Cystic fibrosis transmembrane conductance regulator | P13569 | Tezacaftor CHEMBL3544914 | 446 |
| Ion channel | Neuronal acetylcholine receptor subunit alpha-7 | P36544 | Amobarbital CHEMBL267894 | 13 |
| Ion channel | Neuronal acetylcholine receptor subunit beta-4 | P30926 | Dextromethorphan CHEMBL52440 | 12 |
| Ion channel | Potassium voltage-gated channel subfamily H member 2 | Q12809 | amiodarone CHEMBL633 | 14 |
| Ion channel | Potassium voltage-gated channel subfamily H member 2 | Q12809 | Carvedilol CHEMBL723 | 5 |
| Ion channel | Sodium channel protein type 4 subunit alpha | P35499 | Lidocaine CHEMBL79 | 9 |
| Ion channel | Sodium channel protein type 5 subunit alpha | Q14524 | Carbamazepine CHEMBL108 | 357 |
| Ion channel | Vanilloid receptor | Q8NER1 | Aspartame CHEMBL171679 | 464 |
| Ion channel | Voltage-dependent T-type calcium channel subunit alpha-1G | O43497 | Cinnarizine CHEMBL43064 | 32 |
| Nuclear receptor | Androgen receptor | P10275 | Fluoxymesterone Chembl_377718 CHEMBL1445 | 4 |
| Nuclear receptor | Androgen receptor | P10275 | Levonorgestrel CHEMBL1389 | 10 |
| Nuclear receptor | Androgen receptor | P10275 | Enzalutamide CHEMBL1082407 | 20 |
| Nuclear receptor | Estrogen receptor alpha | P03372 | Clomifene CHEMBL954 | 2 |
| Nuclear receptor | Estrogen receptor alpha | P03372 | Diethylstilbestrol CHEMBL411 | 1 |
| Nuclear receptor | Estrogen receptor beta | Q92731 | Tamoxifen CHEMBL83 | 6 |
| Nuclear receptor | Estrogen receptor beta | Q92731 | Bazedoxifene CHEMBL46740 | 2 |
| Nuclear receptor | Peroxisome proliferator activated receptor alpha | Q07869 | Pioglitazone CHEMBL595 | 208 |
| Nuclear receptor | Peroxisome proliferator-activated receptor gamma | P37231 | Bezafibrate CHEMBL264374 | 15 |
| Nuclear receptor | Peroxisome proliferator-activated receptor gamma | P37231 | Rosiglitazone CHEMBL121 | 252 |
| Enzyme | Carbonic anhydrase IX | Q16790 | Zonisamide CHEMBL750 | 2 |
| Enzyme | DNA topoisomerase II alpha | P11388 | Ciprofloxacin CHEMBL8 | 120 |
| Enzyme | Epidermal growth factor receptor erbB1 | P00533 | Afatinib CHEMBL1173655 | 1 |
| Enzyme | Platelet-derived growth factor receptor beta | P09619 | Imatinib CHEMBL941 | 47 |
| Enzyme | Platelet-derived growth factor receptor beta | P09619 | Sunitinib CHEMBL535 | 14 |
| Enzyme | Prostaglandin G/H synthase 1 | P23219 | Diclofenac CHEMBL139 | 16 |
| Enzyme | Prostaglandin G/H synthase 1 | P23219 | Ibuprofen CHEMBL521 | 3 |
| Enzyme | Prostaglandin G/H synthase 2 | P35354 | Indomethacin CHEMBL6 | 2 |
| Enzyme | Serine/threonine-protein kinase pim-1 | P11309 | Adenosine monophosphate CHEMBL752 | 49 |
| Enzyme | Vascular endothelial growth factor receptor 3 | P35916 | Sorafenib CHEMBL1336 | 6 |

**Reference**

1. Favoni, R.E. and de Cupis, A. (1998) Steroidal and nonsteroidal oestrogen antagonists in breast cancer: basic and clinical appraisal. *Trends Pharmacol Sci*, **19**, 406-415.

2. de Medina, P., Favre, G. and Poirot, M. (2004) Multiple targeting by the antitumor drug tamoxifen: a structure-activity study. *Curr Med Chem Anticancer Agents*, **4**, 491-508.

3. Hertz, D.L., McLeod, H.L. and Irvin, W.J., Jr. (2012) Tamoxifen and CYP2D6: a contradiction of data. *Oncologist*, **17**, 620-630.

4. Janvilisri, T., Venter, H., Shahi, S., Reuter, G., Balakrishnan, L. and van Veen, H.W. (2003) Sterol transport by the human breast cancer resistance protein (ABCG2) expressed in Lactococcus lactis. *J Biol Chem*, **278**, 20645-20651.

5. Zhou, S.F., Zhou, Z.W., Yang, L.P. and Cai, J.P. (2009) Substrates, inducers, inhibitors and structure-activity relationships of human Cytochrome P450 2C9 and implications in drug development. *Curr Med Chem*, **16**, 3480-3675.

6. Kiyotani, K., Mushiroda, T., Imamura, C.K., Hosono, N., Tsunoda, T., Kubo, M., Tanigawara, Y., Flockhart, D.A., Desta, Z., Skaar, T.C. *et al.* (2010) Significant effect of polymorphisms in CYP2D6 and ABCC2 on clinical outcomes of adjuvant tamoxifen therapy for breast cancer patients. *J Clin Oncol*, **28**, 1287-1293.

7. Riley, J., Styles, J., Verschoyle, R.D., Stanley, L.A., White, I.N. and Gant, T.W. (2000) Association of tamoxifen biliary excretion rate with prior tamoxifen exposure and increased mdr1b expression. *Biochem Pharmacol*, **60**, 233-239.

8. Wang, E.J., Casciano, C.N., Clement, R.P. and Johnson, W.W. (2003) Fluorescent substrates of sister-P-glycoprotein (BSEP) evaluated as markers of active transport and inhibition: evidence for contingent unequal binding sites. *Pharm Res*, **20**, 537-544.

9. Paul, R., Silve, S., De Nys, N., Dupuy, P.H., Bouteiller, C.L., Rosenfeld, J., Ferrara, P., Le Fur, G., Casellas, P. and Loison, G. (1998) Both the immunosuppressant SR31747 and the antiestrogen tamoxifen bind to an emopamil-insensitive site of mammalian Delta8-Delta7 sterol isomerase. *J Pharmacol Exp Ther*, **285**, 1296-1302.

10. O'Brian, C.A., Liskamp, R.M., Solomon, D.H. and Weinstein, I.B. (1985) Inhibition of protein kinase C by tamoxifen. *Cancer Res*, **45**, 2462-2465.

11. Fleming, C.D., Bencharit, S., Edwards, C.C., Hyatt, J.L., Tsurkan, L., Bai, F., Fraga, C., Morton, C.L., Howard-Williams, E.L., Potter, P.M. *et al.* (2005) Structural insights into drug processing by human carboxylesterase 1: tamoxifen, mevastatin, and inhibition by benzil. *J Mol Biol*, **352**, 165-177.

12. Sun, D., Sharma, A.K., Dellinger, R.W., Blevins-Primeau, A.S., Balliet, R.M., Chen, G., Boyiri, T., Amin, S. and Lazarus, P. (2007) Glucuronidation of active tamoxifen metabolites by the human UDP glucuronosyltransferases. *Drug Metab Dispos*, **35**, 2006-2014.

13. Preissner, S., Kroll, K., Dunkel, M., Senger, C., Goldsobel, G., Kuzman, D., Guenther, S., Winnenburg, R., Schroeder, M. and Preissner, R. (2010) SuperCYP: a comprehensive database on Cytochrome P450 enzymes including a tool for analysis of CYP-drug interactions. *Nucleic Acids Res*, **38**, D237-243.

14. Crewe, H.K., Notley, L.M., Wunsch, R.M., Lennard, M.S. and Gillam, E.M. (2002) Metabolism of tamoxifen by recombinant human cytochrome P450 enzymes: formation of the 4-hydroxy, 4'-hydroxy and N-desmethyl metabolites and isomerization of trans-4-hydroxytamoxifen. *Drug Metab Dispos*, **30**, 869-874.

15. Desta, Z., Ward, B.A., Soukhova, N.V. and Flockhart, D.A. (2004) Comprehensive evaluation of tamoxifen sequential biotransformation by the human cytochrome P450 system in vitro: prominent roles for CYP3A and CYP2D6. *J Pharmacol Exp Ther*, **310**, 1062-1075.

16. Walsky, R.L., Gaman, E.A. and Obach, R.S. (2005) Examination of 209 drugs for inhibition of cytochrome P450 2C8. *J Clin Pharmacol*, **45**, 68-78.

17. Higgins, M.J. and Stearns, V. (2010) CYP2D6 polymorphisms and tamoxifen metabolism: clinical relevance. *Curr Oncol Rep*, **12**, 7-15.

18. Williams, J.A., Ring, B.J., Cantrell, V.E., Jones, D.R., Eckstein, J., Ruterbories, K., Hamman, M.A., Hall, S.D. and Wrighton, S.A. (2002) Comparative metabolic capabilities of CYP3A4, CYP3A5, and CYP3A7. *Drug Metab Dispos*, **30**, 883-891.

19. Santner, S.J. and Santen, R.J. (1993) Inhibition of estrone sulfatase and 17 beta-hydroxysteroid dehydrogenase by antiestrogens. *J Steroid Biochem Mol Biol*, **45**, 383-390.

20. Levine, R.M., Rubalcaba, E., Lippman, M.E. and Cowan, K.H. (1985) Effects of estrogen and tamoxifen on the regulation of dihydrofolate reductase gene expression in a human breast cancer cell line. *Cancer Res*, **45**, 1644-1650.

21. Nuwaysir, E.F., Daggett, D.A., Jordan, V.C. and Pitot, H.C. (1996) Phase II enzyme expression in rat liver in response to the antiestrogen tamoxifen. *Cancer Res*, **56**, 3704-3710.

22. Ritchie, G.A. (1980) The direct inhibition of prostaglandin synthetase of human breast cancer tumor tissue by tamoxifen. *Recent Results Cancer Res*, **71**, 96-101.

23. Nilsson, U.W., Garvin, S. and Dabrosin, C. (2007) MMP-2 and MMP-9 activity is regulated by estradiol and tamoxifen in cultured human breast cancer cells. *Breast Cancer Res Treat*, **102**, 253-261.

24. Lax, E.R., Rumstadt, F., Plasczyk, H., Peetz, A. and Schriefers, H. (1983) Antagonistic action of estrogens, flutamide, and human growth hormone on androgen-induced changes in the activities of some enzymes of hepatic steroid metabolism in the rat. *Endocrinology*, **113**, 1043-1055.

25. Messiha, F.S. (1990) Leu-enkephalin, tamoxifen and ethanol interactions: effects on motility and hepatic ethanol metabolizing enzymes. *Gen Pharmacol*, **21**, 45-48.

26. Paavonen, T., Aronen, H., Pyrhonen, S., Hajba, A. and Andersson, L.C. (1991) The effect of toremifene therapy on serum immunoglobulin levels in breast cancer. *APMIS*, **99**, 849-853.
